# Performance of Routine MRI Reporting for Parapharyngeal Space Tumors: A Retrospective Radiologic–Pathologic Comparison

**DOI:** 10.3390/jcm15010392

**Published:** 2026-01-05

**Authors:** Mohammed Alshahrani, Mohammed Almayouf, Aseel Doubi, Omar Alotaibi, Sharif Almatrafi, Khalid AlQahtani, Saleh Aldhahri, Majed Albarrak, Mohammed Alessa, Ahmed Albosaily, Faisal Alzahrani

**Affiliations:** 1 Department of Otolaryngology—Head and Neck Surgery, King Fahad Medical City, Riyadh 11525, Saudi Arabia; moalmayouf@kfmc.med.sa (M.A.); omar3alotaibi@gmail.com (O.A.); majed.hns@gmail.com (M.A.); 2 Department of Otolaryngology—Head and Neck Surgery, King Saud Medical City, Riyadh 11525, Saudi Arabia; aodoubi@gmail.com (A.D.); dr.albosaily@hotmail.com (A.A.); 3 Department of Otorhinolaryngology—Head and Neck Surgery, College of Medicine, Prince Sattam Bin Abdulaziz University, Alkharj 11942, Saudi Arabia; sharifalmatrafi@gmail.com; 4 Department of Otolaryngology—Head and Neck Surgery, College of Medicine, King Saud University, Riyadh 11461, Saudi Arabia; kalqahtani@ksu.edu.sa (K.A.); saldhahri@ksu.edu.sa (S.A.); maalessa@ksu.edu.sa (M.A.); fralzahrani@ksu.edu.sa (F.A.)

**Keywords:** magnetic resonance imaging, parapharyngeal space, head and neck neoplasms, diagnostic imaging, retrospective study

## Abstract

**Background/Objectives:** The parapharyngeal space is a complex anatomical region that houses critical neurovascular structures and serves as the origin of rare tumors, which account for 0.5–1% of head and neck neoplasms. Magnetic resonance imaging (MRI) is useful for their preoperative assessment. However, its accuracy in real-world clinical settings remains underexplored. This study aimed to investigate the diagnostic accuracy of MRI for parapharyngeal tumors at two tertiary centers. **Methods:** This retrospective study included patients who underwent MRI and surgical excision at two tertiary centers in Saudi Arabia between 2018 and 2024. Two reviewers independently extracted their MRI data and compared them with the final pathological data to determine the diagnostic performance of MRI. **Results:** Of the 31 patients (58.1% female; median age, 37.5 years), 90.3% had benign tumors. Neurogenic (41.9%) and salivary (25.8%) tumors were most common; 61.3% were located within the pre-styloid space. The benign and malignant groups had comparable baseline characteristics. MRI demonstrated moderate overall diagnostic agreement (κ = 0.525) and near-perfect concordance for schwannomas (κ = 0.912) and paragangliomas (κ = 0.839) but poor agreement for hemangiopericytomas (κ = −0.051). It had high accuracy (90.3%), specificity (92.9%), and negative predictive value (96.3%) for detecting malignancy but limited sensitivity (66.7%) or positive predictive value (50.0%). Nonetheless, cautious interpretation is required due to the limited prevalence of malignancy in the cohort (*n* = 3). **Conclusions:** MRI demonstrated high specificity for benign parapharyngeal space lesions in routine clinical reporting within this retrospective cohort, reflecting strong radiologic–pathologic agreement. Estimates of sensitivity and positive predictive value for malignancy were influenced by the limited number of malignant cases. Accordingly, the reported diagnostic performance measures should be interpreted as descriptive and exploratory, characterizing real-world MRI performance rather than definitive diagnostic accuracy.

## 1. Introduction

The parapharyngeal space (PPS) is an inverted pyramid-shaped area located in the neck. As it contains several vital nerves and blood vessels, accurate assessment of tumors originating from this region is crucial [1,2]. Tumors in this space are uncommon, accounting for 0.5–1% of all head and neck tumors; most (70–80%) are benign [3,4,5].

The most frequent pathologies include salivary gland tumors, especially pleomorphic adenomas, and neurogenic tumors such as schwannomas and paragangliomas [3,4,6]. Malignant tumors found in this area can include acinic cell carcinoma, myoepithelial carcinoma, and chondroid chordoma [7,8]. Accurate imaging interpretation is crucial for defining tumor boundaries and relationships with vital structures, because misinterpretation can increase the risk of incomplete resection or complications such as hemorrhage and nerve injury [9,10]. Magnetic resonance imaging (MRI) offers superior soft-tissue contrast over computed tomography (CT) and plays a pivotal role in the evaluation of PPS tumors [2,11,12]. It facilitates the assessment of tumor size, extent, and invasion of adjacent compartments or vital structures such as the carotid artery or cranial nerves. It also provides vital information for staging and surgical approach selection, including the feasibility of minimally invasive techniques [5,10,13].

MRI also facilitates monitoring of therapeutic response, assessment for residual disease, detection of postoperative complications, and guidance for subsequent management strategies [14,15]. Its non-ionizing nature makes MRI preferable for repeated imaging, especially in pediatric patients requiring long-term follow-up. However, MRI is susceptible to motion artifacts and interference from dental hardware, which can degrade image quality and obscure lesions [2,16]. Furthermore, overlapping MRI signals among histologically distinct tumors can lead to diagnostic ambiguity [17].

The MRI features of several tumor types have been described. Pleomorphic adenomas are typically hyperintense on T2-weighted images. Schwannomas show homogeneous enhancement, and paragangliomas have a classic “salt-and-pepper” pattern with flow voids [2,18,19]. The reported diagnostic accuracy of MRI is 80–95%, especially with advanced imaging techniques such as diffusion-weighted imaging (DWI) or perfusion analysis [2,18]. However, these results are obtained from controlled research environments or focused imaging analyses and may not reflect everyday clinical conditions.

Despite extensive descriptions of the imaging characteristics of parapharyngeal space tumors, there remains limited evidence on how accurately routine clinical MRI reports, as interpreted in daily practice, correspond to final histopathological diagnoses. Most prior studies assess imaging features under controlled or protocol-driven conditions, which may not reflect real-world reporting behavior, particularly in rare tumors with heterogeneous presentation. In this context, the present study aimed to evaluate the agreement between routine preoperative MRI reports and definitive histopathology for parapharyngeal space tumors at two tertiary centers, with a secondary focus on the descriptive performance of MRI in distinguishing benign from malignant lesions. The null hypothesis was that routine clinical MRI reporting does not demonstrate agreement with final histopathology beyond chance in the classification of parapharyngeal space tumors.

## 2. Materials and Methods

### 2.1. Study Design and Participants

This study involved the retrospective review of patient charts to evaluate the accuracy of MRI for diagnosing PPS tumors. We identified patients who were treated for this condition at two major hospitals in Riyadh, Saudi Arabia, between January 2018 and December 2024. Ethical approval for this research was obtained from the Institutional Review Board of King Fahad Medical City, Riyadh, Saudi Arabia (IRB Log Number: 25-523) and waived the requirement for informed consent due to the retrospective design of the study. This study was conducted in accordance with the ethical principles outlined in the Declaration of Helsinki. Reporting followed the STARD checklist for diagnostic test accuracy studies [20].

### 2.2. Eligibility Criteria

The inclusion criteria were as follows: patients with a primary parapharyngeal tumor who underwent a complete diagnostic workup (including institutional MRI) and surgical excision by a head and neck oncology surgeon. Patients were excluded if they (a) were treated partially at another institution, (b) did not have MRI as part of their workup, (c) had their MRI performed at an outside facility or reported by a non-head and neck subspecialist radiologist, or (d) were diagnosed with other primary head and neck tumors that presented with a parapharyngeal mass and were subsequently identified as metastatic lymph nodes. The exclusion of externally performed MRI studies was intended to ensure protocol consistency but may introduce institutional bias and limit external validity.

### 2.3. Index Test: Magnetic Resonance Imaging

All patients underwent standardized neck MRI using 1.5-T or 3-T MRI scanners. The scans included axial T1-weighted, axial T2-weighted, axial fat-suppressed T2-weighted, and post-contrast T1-weighted sequences in the axial, coronal, and sagittal planes. The preoperative MRI reports documented in the electronic medical records were used as the index tests. These reports were prepared by radiologists with subspecialty training in head and neck imaging. The radiologists were aware of the clinical history of the patients but were blinded to the final histopathological outcomes at the time of the initial report.

### 2.4. Reference Standard: Histopathology

The reference standard was the final histopathological diagnosis based on the analysis of the surgically excised tumor specimen. All specimens were processed and evaluated by board-certified pathologists in accordance with standard laboratory procedures. The pathologists were aware of the preoperative radiological diagnosis as part of the clinical information provided. However, they performed their evaluations independently.

### 2.5. Data Extraction and Interpretation

The data were extracted independently by two reviewers to minimize errors and bias. Both reviewers were otolaryngology researchers with prior experience in retrospective data abstraction and study design. The MRI reports were prepared and interpreted by fellowship-trained head and neck radiologists with several years of post-training experience in diagnostic imaging. Discrepancies were resolved through consensus with a senior researcher. The primary radiological diagnosis reported in the MRI was compared with the final pathological diagnosis and categorized as follows: (a) Correct: the top differential diagnosis in the MRI report exactly matched the final histopathological diagnosis (e.g., MRI reported “schwannoma” and pathology confirmed schwannoma); (b) Broad diagnosis: The MRI diagnosis was accurate but non-specific, providing a correct general category without specifying the exact pathology (e.g., MRI reported “benign neurogenic tumor” and pathology confirmed neurofibroma; or MRI reported “salivary gland neoplasm” and pathology revealed pleomorphic adenoma). This category reflects clinically appropriate diagnostic caution where MRI features support a pathological family but not a specific entity; and (c) Incorrect: Either: (i) the top differential diagnosis was wrong (e.g., MRI reported “pleomorphic adenoma” but pathology showed paraganglioma); OR (ii) the report was inconclusive, defined as providing completely non-committal language (e.g., “indeterminate mass, differential includes both benign and malignant entities, further tissue diagnosis recommended”) or listing multiple divergent possibilities without ranking them. These categories were used for descriptive comparison and are not validated diagnostic accuracy measures. Radiologists were not blinded to clinical information, and pathologists were aware of imaging findings, reflecting real-world clinical practice but limiting strict diagnostic accuracy inference.

### 2.6. Outcomes

The primary outcome of this study was the diagnostic performance of MRI in differentiating benign from malignant PPS tumors. This was expressed in terms of sensitivity, specificity, positive and negative predictive values, and overall accuracy.

The secondary outcomes were as follows: (a) the level of agreement between the MRI diagnosis and final histopathological findings for specific tumor types (quantified using Fleiss’ κ coefficient); and (b) comparison of the demographic and tumor characteristics of the benign and malignant groups.

### 2.7. Statistical Analysis

Statistical analyses were performed using R software (version 4.3.3). Statistical significance was set at *p* < 0.05. All statistical analyses were conducted under the supervision of a biostatistician with expertise in diagnostic accuracy and agreement studies. The key characteristics of the patients and their tumors were summarized using descriptive statistics. The diagnostic performance of MRI in distinguishing between benign and malignant lesions was evaluated by calculating the sensitivity, specificity, positive predictive value (PPV), negative predictive value (NPV), and overall accuracy. The agreement between the MRI diagnoses and the final histopathology results was assessed using the Fleiss’ Kappa (κ) coefficient, and the results were interpreted based on the Landis and Koch criteria. The benign and malignant groups were compared using appropriate statistical tests for the different data types. The two-sample *t*-test was used to compare normally distributed continuous variables (such as age and tumor size), while the Wilcoxon rank-sum test was used for those that were not. Fisher’s exact test was used to compare categorical variables (e.g., sex, tumor type, and location) because of the small sample size and low expected cell frequencies. All eligible patients during the study period were included. Therefore, no sample size calculations or power analysis were performed. This approach is consistent with those of similar retrospective diagnostic accuracy studies of rare tumors, where the available number of cases determines the sample size.

## 3. Results

### 3.1. Patient and Tumor Characteristics

The data of 31 patients who underwent surgery for PPS tumors were used for the final analysis. Eighteen (58.1%) and thirteen (41.9%) of the participants were females and males, respectively. The median age was 37.5 years (interquartile range: 24.8–51.0 years). The median tumor size was 5.0 cm (interquartile range: 4.0–5.6 cm). The final histopathological analysis confirmed that 28 (90.3%) and 3 (9.7%) tumors were benign and malignant, respectively. Neurogenic tumors were the most common pathology, accounting for 13 cases (41.9%), followed by salivary tumors (n = 8, 25.8%). Nineteen (61.3%) and twelve (38.7%) tumors were located in the pre-styloid and post-styloid spaces, respectively. The characteristics of the benign and malignant tumors were not significantly different. No significant associations were observed between malignancy and patient gender (*p* = 1.000), tumor type (*p* = 0.367), or anatomical site (reptiloid vs. post styloid, *p* = 1.000). Table 1 presents a comprehensive overview of the baseline characteristics.

### 3.2. Diagnostic Agreement

The overall agreement between the primary MRI diagnosis and the final histopathological diagnosis was moderate (Fleiss’ κ = 0.525; *p* < 0.001). The level of agreement varied substantially across the tumor types (Table 2). It was almost perfect for schwannoma (κ = 0.912) and paraganglioma (κ = 0.839) and perfect for branchial pouch cyst and lipoma (κ = 1.000), indicating reliable MRI-based identification of these specific entities. Substantial agreement was also observed for common pathologies such as neurofibroma (κ = 0.631) and pleomorphic adenoma (κ = 0.610). However, no statistically significant agreement was observed for several other pathologies, including hemangiopericytoma (κ = −0.051) and Warthin tumor (κ = −0.016), suggesting that their imaging features were not reliably distinguishable from those of other entities.

### 3.3. Diagnostic Performance for Malignancy

Table 3 summarizes information on the performance of MRI in distinguishing between benign and malignant lesions. The overall diagnostic accuracy was 90.3% (95% confidence interval [CI]: 74.2–97.9%). MRI demonstrated high specificity (92.9%, 95% CI: 76.5–99.1%) and a high negative predictive value (96.3%, 95% CI: 81.0–99.9%), indicating that it is very effective at correctly identifying benign lesions, and the probability of malignancy is low when MRI findings suggest a benign lesion. The sensitivity for detecting malignancy or classifying the lesion as inconclusive for further investigation was 66.7% (95% CI: 9.4–99.2%), and the positive predictive value was 50.0% (95% CI: 6.8–93.2%); such a wide confidence interval stems from the low prevalence of malignancy in the cohort (n = 3).

## 4. Discussion

This study aimed to evaluate the performance of routine clinical MRI reporting and its level of agreement with pathologic findings for parapharyngeal space tumors. The findings demonstrate the high specificity of MRI for distinguishing benign from malignant lesions but its limited sensitivity for detecting malignancy. These findings confirm our initial hypothesis that MRI is reliable for identifying tumors with characteristic imaging features but less effective when the imaging characteristics of benign and malignant entities overlap. The study achieved its objective of quantifying the real-world diagnostic performance of MRI and highlighting the tumor types for which MRI can be used as a stand-alone diagnostic tool and those for which further confirmatory testing is required.

Our study confirms the role of MRI in the evaluation of PPS tumors, consistent with its established reputation for soft tissue assessment [4,21]. The overall diagnostic accuracy of 90.3% for our cohort aligns with the accuracy rates of 80–96% reported in previous studies [18,22]. Our finding of high specificity (92.9%) is similar to those of Stoia et al. (2025) and Vargas et al. (2022), who reported specificities of 97–100% [21,23]. This suggests that MRI is reliable for ruling out malignancy; a lesion with benign features on MRI is very likely to be benign. However, our sensitivity (66.7%) and positive predictive value (50%) were lower than the sensitivity (80–90.4%) and positive predictive value (80–93.4%) reported in other series [21,22]. This discrepancy can largely be explained by the low prevalence of malignancy among PPS tumors. Our observation that 90% of the tumors were benign is consistent with previous reports (70–96%) [23,24]. The positive predictive value is susceptible to the impact of false positives when the prevalence of a condition is low, as with malignancy (n = 3) in this study. The situation is further complicated by the overlapping imaging characteristics of some benign and malignant tumors. Inflammatory reactions or atypical features in benign lesions can lead to false-positive interpretations [21,22].

The variability in diagnostic agreement observed in our study is likely attributable to the presence or absence of pathognomonic imaging features for specific tumor types. The excellent agreement for lipomas, schwannomas, and paragangliomas is attributable to their distinct MRI characteristics. Lipomas are reliably identified based on their homogeneous signal intensity, which resembles that of subcutaneous fat on all sequences and is suppressed by fat-saturation techniques [25,26]. Schwannomas are distinguished by their intense, homogeneous enhancement and lack of “flow voids”, whereas paragangliomas are characterized by hypervascularity, which creates the classic “salt-and-pepper” appearance [18,26,27]. The agreement level for pleomorphic adenomas is attributable to their typical presentation as well-defined, T2-hyperintense masses in the reptiloid space. This site specificity simplifies diagnosis [4,19].

The poor agreement for other pathologies, such as hemangiopericytoma and Warthin’s tumor, highlights the limitations of MRI in the absence of classic signs. Hemangiopericytomas are vascular but lack specific imaging findings and can mimic other neoplasms, such as pleomorphic adenomas. They often require angiography for definitive diagnosis [28,29]. Warthin’s tumor is a well-known mimicker of malignancy, and its overlapping features on ADC mapping and dynamic contrast-enhanced MRI complicate its differentiation from malignant lesions [21,30]. Therefore, the diagnostic accuracy of MRI is not uniform; it is good for identifying tumors with well-defined radiological signatures but less reliable for those with overlapping imaging characteristics. Diagnostic accuracy can be improved by incorporating other modalities, such as CT or angiography, for inconclusive cases [18,27].

MRI is indispensable for the preoperative assessment of PPS tumors. Its superior soft tissue resolution enables precise delineation of tumor boundaries and relationships to neurovascular structures. This is essential for selecting the appropriate surgical approach, from transcervical for inferior lesions to more extensive transmandibular or skull-base approaches for superior or suspected malignant tumors [18,31]. The anatomical information provided by MRI and its non-ionizing nature are fundamental in determining the overall management strategy when considering a “wait-and-scan” approach. Active surveillance is a viable option for selected patients, such as older adults and those with asymptomatic neurogenic lesions for whom surgery carries a high risk of nerve damage [4,32]. This is because most PPS tumors are benign and indolent. The decision to adopt this conservative management approach is supported by evidence showing that many tumors remain stable or even regress. It also relies heavily on the confidence in a benign diagnosis based on MRI [4].

This study has several strengths. It is based on real-world clinical data from two tertiary centers, and these reflect the diagnostic challenges faced by clinicians. The use of a two-reviewer system for data extraction enhance the reliability of our findings. The detailed Kappa analysis provides a nuanced view of diagnostic performance that a single accuracy metric cannot capture. Our findings have direct clinical relevance, highlighting the scenarios in which MRI findings can contribute meaningfully to management and those that require further diagnostic procedures such as fine-needle aspiration or angiography. This work also has educational value because it identifies tumor types with reliable MRI features and those that present interpretive challenges, helping guide radiologists and surgeons toward more informed diagnostic decisions. However, this study also has certain limitations. The primary limitation is the small sample size, which is common in studies of rare pathologies such as PPS tumors. The very low number of malignant cases limited the statistical power for assessing malignancy-related metrics and resulted in wide confidence intervals that necessitate cautious interpretation. Moreover, radiologists who reviewed MRI images were fellowship-trained and had several years of experience, which may not translate well to other settings. Large prospective multi-center studies are needed to build a robust dataset for malignant tumors and enable more generalizable conclusions. Future research should include standardized, blinded re-reviews of images to examine the added value of other sequences, such as DWI and perfusion, in differentiating tumors with poor agreement in our study.

## 5. Conclusions

This retrospective study demonstrates that routine MRI reporting achieves high specificity for benign parapharyngeal space tumors, supporting robust radiologic–pathologic agreement in clinical practice. Sensitivity for malignant disease was lower, largely attributable to the low prevalence of malignancy within the cohort. Therefore, the diagnostic performance metrics presented are descriptive and exploratory and should be interpreted in the context of the study design rather than as confirmatory measures of diagnostic accuracy. Overall, the findings highlight the value of MRI in the routine evaluation of parapharyngeal space tumors while underscoring the need for larger, multi-institutional studies to further refine diagnostic criteria for malignancy routine MRI reporting demonstrated high specificity for benign parapharyngeal space tumors. However, our data showed a limited sensitivity for detecting malignant diseases, primarily due to the low prevalence of malignant cases. Using other imaging modalities alongside MRI might help to improve accuracy in non-typical tumors. Larger multi-institutional cohorts studies are needed to refine the diagnostic criteria of benign and malignant parapharyngeal space tumors and overcome the low prevalence of malignancy in our cohort.

## Figures and Tables

**Table 1:** Baseline characteristics for patients with PPS tumors.

Baseline Variable		Histology		Test
		Benign	Malignant	
Age	Min/Max	7.0/65.0	26.0/61.0	*p*-value: 0.6813 (Two sample *t*-test)
	Med [IQR]	37.5 [24.8; 51.0]	38.0 [32.0; 49.5]	
	Mean (std)	37.5 (16.6)	41.7 (17.8)	
	N	28	3	
Gender	F	16 (88.89%)	2 (11.11%)	*p*-value: 1.0000 (Fisher’s exact test for count data)
	M	12 (92.31%)	1 (7.69%)	
Size	Min/Max	2.0/6.5	3.5/8.0	*p*-value: 0.5872 (Wilcoxon rank sum test)
	Med [IQR]	5.0 [4.0; 5.6]	3.5 [3.5; 5.8]	
	Mean (std)	4.8 (1.1)	5.0 (2.6)	
	N	28	3	
Type	Congenital cysts	1 (100.00%)	0 (0%)	*p*-value: 0.3666 (Fisher’s exact test for count data)
	Lipoma	2 (100.00%)	0 (0%)	
	Neurogenic tumor	12 (92.31%)	1 (7.69%)	
	Paraganglioma	3 (100.00%)	0 (0%)	
	Salivary tumor	7 (87.50%)	1 (12.50%)	
	Sarcoma	0 (0%)	1 (100.00%)	
	Vascular tumor	3 (100.00%)	0 (0%)	
Site	Post-styloid	11 (91.67%)	1 (8.33%)	*p*-value: 1.0000 (Fisher’s exact test for count data)
	Pre-styloid	17 (89.47%)	2 (10.53%)	

**Table 2:** Agreement between MRI diagnosis and final histopathology.

Diagnosis	No.	Kappa (κ)	Strength of Agreement	z-Value	*p*-Value
Branchial pouch cyst *	1	1.000	Perfect	5.57	<0.001
Lipoma *	2	1.000	Perfect	5.57	<0.001
Schwannoma	8	0.912	Almost Perfect	5.08	<0.001
Paraganglioma	4	0.839	Almost Perfect	4.67	<0.001
Neurofibroma *	3	0.631	Substantial	3.51	<0.001
Pleomorphic adenoma	6	0.610	Substantial	3.40	<0.001
Other pathologies ^1^	7	<0	Poor	-	>0.05

This table details the inter-rater agreement for specific diagnoses. The overall agreement was moderate (Fleiss’ κ = 0.525, z = 8.6, *p* < 0.001). * Estimates were based on less than four cases. ^1^ Includes diagnoses such as hemangiopericytoma, Warthin tumor, and ganglioneuroma, for which no statistically significant agreement was observed.

**Table 3:** Diagnostic performance of MRI for differentiating benign from malignant lesions.

	Pathology: Malignant	Pathology: Benign
MRI: Malignant/inconclusive	2	2
MRI: Benign	1	26
**Performance Metric**	**Value (%)**	**95% Confidence Interval**
Sensitivity *	66.7%	9.4–99.2%
Specificity	92.9%	76.5–99.1%
Positive predictive value (PPV) *	50.0%	6.8–93.2%
Negative predictive value (NPV)	96.3%	81.0–99.9%
Overall accuracy	90.3%	74.2–98.0%

This table summarizes the performance of MRI based on the following contingency table. * Unstable estimates, interpret with caution.

## Data Availability

The original contributions presented in this study are included in the article. Further inquiries can be directed to the corresponding author.

## Abbreviations

The following abbreviations are used in this manuscript:

CT	Computed tomography
DWI	Diffusion-weighted imaging
NPV	Negative predictive value
MRI	Magnetic resonance imaging
PPV	Positive predictive value
PPS	Parapharyngeal space
